# Fabric-Based Flexible Pressure Sensor Arrays with Ultra-Wide Pressure Range for Lower Limb Motion Capture System

**DOI:** 10.34133/research.0835

**Published:** 2025-08-18

**Authors:** Xiaohua Wu, Yuxuan Liang, Longsheng Lu, Shu Yang, Zhanbo Liang, Feilong Liu, Xiaoyu Lu, Bowen Xiao, Yilin Zhong, Yingxi Xie

**Affiliations:** School of Mechanical & Automotive Engineering, South China University of Technology, Guangzhou 510641, China.

## Abstract

The lower limb motion capture technology has garnered significant attention as a pivotal enabler in extended reality, sports science, film production, and medical rehabilitation. However, existing mature systems face critical challenges, including restricted operational environments, interference with natural activities, and cumbersome wearability. Here, a flexible insole pressure sensor array with an ultra-wide sensing range is developed using dip coating, laser cutting, and hot pressing, enabling lower limb motion capture. The developed fabric-based flexible pressure sensor exhibited an ultra-wide pressure range (3,770.9 kPa), high sensitivity (2.68 kPa^−1^), rapid response, recovery times (17.2 ms/3.5 ms), and high work life (>4 million loading/unloading cycles). The insole-shaped flexible pressure sensor array accurately measures pressure in different postures, achieving 95.5% classification accuracy across 10 dynamic and static poses. More importantly, the system achieved a joint position prediction accuracy of 7.8 pixels (~3.6 cm) in lower limb pose estimation. This high-precision lower limb motion capture system represents an ideal terminal for future extended reality applications, offering seamless integration, comfortable use, easily wearable design, and broad accessibility.

## Introduction

Flexible pressure sensor arrays, as an emerging sensing technology, show great potential for applications in advanced intelligent human–computer interaction (HCI) [1,2]. As a core component of future digital life, intelligent HCI relies on precise motion capture technology, particularly for lower limb movements, to enable natural interaction and control [3–5]. Flexible pressure sensor arrays offer a novel technological approach toward achieving this goal.

Mainstream lower limb motion capture technologies include optical motion capture systems and inertial sensor suits [6]. Optical motion capture uses multiple cameras to track markers and capture complex movements with high precision [7]. But it is expensive, dependent on specific environments, and prone to occlusion and privacy issues. Inertial motion capture suits [8–10] integrate inertial measurement units into suits at joint-specific locations, providing robust joint position tracking without environmental constraints. Nevertheless, their cumbersome wiring and setup interfere with daily activities. These limitations, including environmental dependency, wearing complexity, and interference with natural movement, make both technologies unsuitable for achieving the seamless socialization and immersive experiences envisioned in intelligent HCI.

Benefiting from advancements in fabrication techniques for conductive and force-sensitive structures [11,12], such as conductive particle doping [13], dip coating [14], templating methods [15], and electrospinning [16], researchers have developed high-performance flexible sensors to overcome the limitations of current solutions for lower-limb motion estimation. For example, Kim et al. [17] utilized 20 flexible strain sensors embedded in suit to detect whole-body joint locations. Similarly, Yang et al. [18] developed highly sensitive flexible strain sensors and deployed 7 sensors at joint locations, achieving whole-body motion capture. Chen et al. [19] integrated 4 flexible strain sensors at the knees and elbows, predicting target joint angles using a bidirectional long short-term memory model. While these approaches eliminate the restrictive nature of inertial suits, they rely on full-body garments, complicating the wearing process and posing challenges for ergonomic and comfortable design due to heavy sensor wiring. To overcome these challenges, researchers have shifted toward insole-based systems for plantar pressure distribution measurement as a proxy for lower limb posture. For instance, Zhang et al. [15] fabricated a pyramid microstructure triboelectric nano-generator insole sensor by template method for identification and privacy protection of 5 users. Zhou et al. [20] developed a flexible pressure sensor with a maximum pressure detection range of 400 kPa by electrospinning and integrated 32 sensors into the insole to distinguish abnormal gait patterns. However, these studies are limited to classifying finite poses, falling short of enabling lower limb movements required for natural interactions in the next-generation Internet.

Here, we propose an ultra-wide flexible pressure sensor array, embedded within an insole, to enable a novel lower limb motion capture system. The developed flexible pressure sensor arrays can measure plantar pressure distributions during dynamic activities, withstanding peak pressures exceeding 3,000 kPa in daily movement [21]. A hollow polyamide (PA) film was introduced between the electrodes and the piezoresistive layers, which served both as an adhesive encapsulation layer and a supporting structure. This design prevents direct contact between the electrodes and the piezoresistive layers, significantly enhancing the tunneling effect and enabling the sensing unit to exhibit higher sensitivity under high pressure. The sensors demonstrate a wide detection range of up to 3,770.9 kPa, high sensitivity of 2.68 kPa^−1^, rapid response, and recovery times of 17.2 and 3.5 ms, respectively, and exceptional durability exceeding 4 million cycles. Additionally, a transformer-based time-series regression model was constructed to process temporal pressure data, enabling precise estimation of lower limb joint positions. This innovative system, designed as an insole, offers a comfortable, nonintrusive, and user-friendly solution for lower limb motion capture. It eliminates interference with natural activities, making it highly suitable for seamless integration into users’ daily lives. This approach not only addresses the limitations of existing motion capture systems but also provides an optimal data input terminal for next-generation Internet applications.

## Results

### Working principle of lower limb motion capture system

**Fig. 1.**
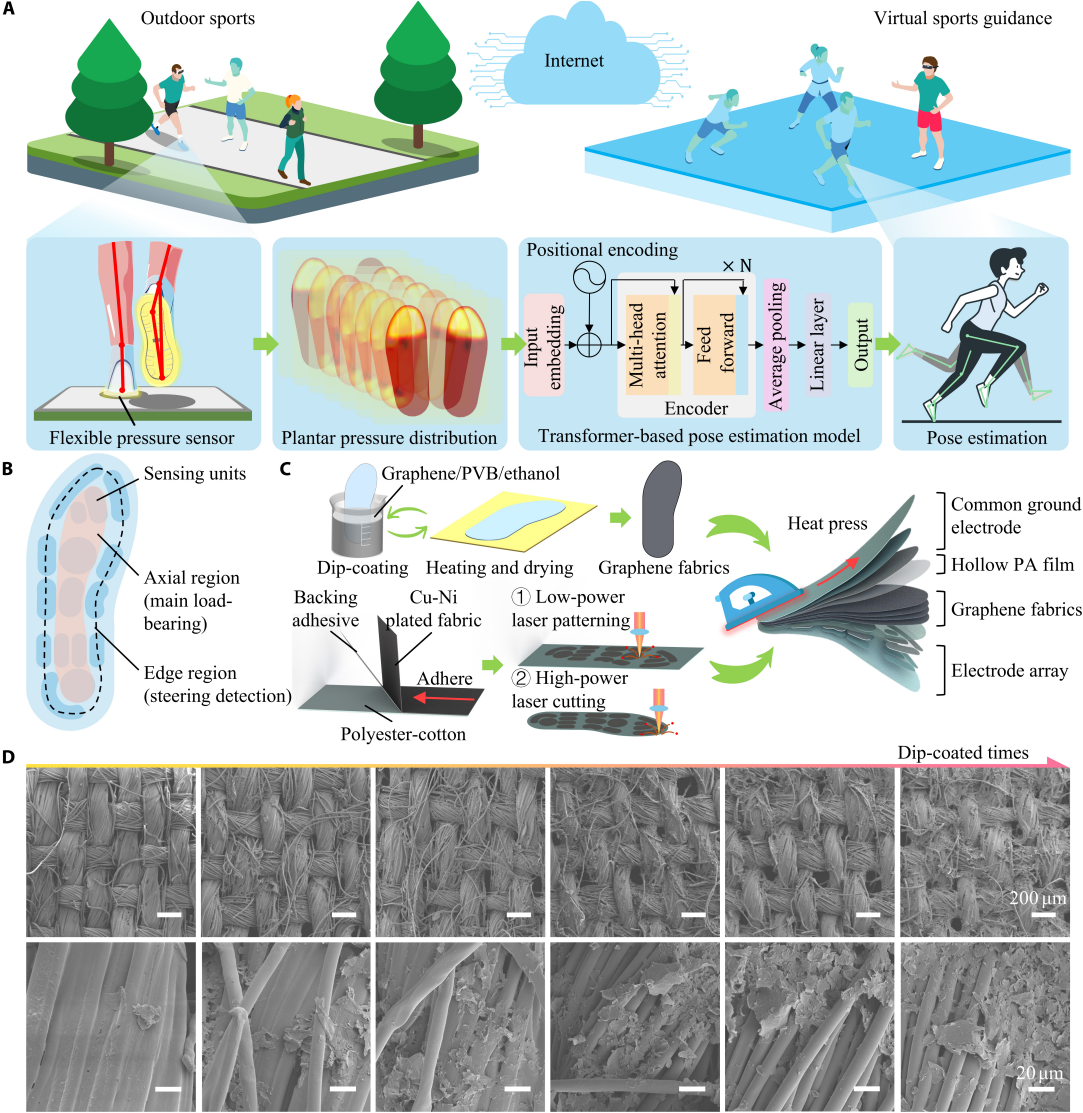
Overview of lower limb motion capture system using fabric-based flexible pressure sensor array. (A) Workflow of the developed lower limb motion capture system. Plantar pressure signals are collected using 2 flexible pressure sensor array insoles. These signals are then processed by a transformer-based regression model to estimate the user’s lower limb joint postures. The estimated postures are rendered within the next-generation Internet, enabling seamless and natural human–computer interaction. (B) Design of the flexible pressure sensor array. The insole-shaped flexible pressure sensor array, designed based on the plantar shape and pressure distribution principles, includes an axial region and an edge region. The axial region detects the primary weight-bearing areas during most postures, and the edge region detects the precise direction of force in most change-of-direction postures. (C) Fabrication process of the flexible pressure sensor array. The sensitive layer was formed by repeatedly dip coating the fabric with a conductive coating, and electrodes were patterned through laser selective cutting. The flexible pressure sensor arrays were fabricated via heat-pressing encapsulation. (D) Microstructural images of polyester cotton samples, ranging from untreated to those subjected to 5 dip-coating cycles.

As shown in Fig. 1A, a flexible pressure sensor array is embedded in the insole. Based on the piezoresistive principle, the sensor array can convert pressure variations into changes in resistance without latency. The shapes of 16 sensing units were designed to conform to the shape of the plantar surface, capturing pressure across different regions and enabling the detection of plantar pressure distribution. The current human posture was estimated by collecting plantar pressure distributions over a period and processing those with a transformer-based time-series deep learning model. Insoles, as an essential everyday item, are better suited than inertial suits or optical capture systems as the ideal motion capture solution for all users in the next-generation Internet (Table S1). By wearing a comfortable insole with flexible pressure sensors that do not interfere with daily activities, users can capture real-time foot pressure during various movements, estimate their posture, and control a digital avatar in the next-generation Internet with accurate, synchronized motions.

The distribution of units in the insole-shaped flexible pressure sensor array was designed based on the plantar shape and pressure distribution principles, as shown in Fig. 1B. When standing still, pressure is focused along the foot’s center. During direction changes, the pressure shifts to the foot’s edge, showing posture changes. Therefore, positioning the sensing units along the plantar axis and contour enables accurate measurement of plantar pressure in different postures, thereby capturing the variations in posture.

To meet user requirements for insoles, the flexible pressure sensor array was made from wear-resistant polyester cotton fabric. As shown in Fig. 1C, the polyester cotton fabric was repeatedly dipped in graphene/polyvinyl butyral (PVB)/ethanol and dried, allowing the PVB to bond graphene to the polyester cotton fibers. This transformed the polyester cotton into graphene fabric with electrical conductivity and piezoresistive properties. The Cu–Ni plated fabric was adhered to the polyester cotton to execute an electrode patterning using low-power and high-power lasers, respectively. The insole-shaped flexible pressure sensor array was assembled using heat-pressing method. During hot pressing, a PA film with hollowed-out patterns served as the encapsulating adhesive. Under external pressure, the contact area between electrodes and graphene fabrics increased, resulting in reduced resistance. Many graphene flakes were brought into close proximity and could conduct current via the tunneling effect, even without direct contact.

As shown in Fig. S1, the tunneling effect mainly occurs between adjacent graphene fabrics and between graphene fabric and the electrode. Dip-coated graphene flakes adhered between the fabric layers; although they did not initially form a conductive network, compression caused them to approach or contact each other, forming stable conductive pathways. The PA film melted during pressing and re-solidified to embed itself between fabric layers, ensuring tight bonding. Its hollowed pattern also separated the electrodes from the graphene fabrics, which enhanced the tunneling effect in this region. The tunneling resistance is commonly expressed as follows [22]:

$$R_{\mathrm{tun}}=\frac{V}{\mathrm{AJ}}=\frac{h^{2}d}{Ae^{2}\sqrt{2\mathrm{m\lambda}}}e^{\frac{4\pi d}{h}\sqrt{2\mathrm{m\lambda}}}$$ (1)

Here, *V* is the applied voltage, *J* is the current density, *A* is the effective contact area, *d* is the barrier width, *λ* is the barrier height, *h* is Planck’s constant, *e* is the elementary charge, and *m* is the effective mass of electrons. This relationship shows that resistance decreases exponentially as the barrier width decreases. The conductance can be calculated by Eq. 2.

$$G=\frac{J}{V}\propto e^{-d}$$ (2)

As pressure increases, the internal distance between conductive elements decreases, reducing the barrier width. Therefore, pressure and barrier width are negatively correlated.

p∝−d (3)

Under constant voltage, the current exhibits a superlinear or even exponential relationship with pressure due to the tunneling effect.

$$\frac{∆I}{I_{0}}\propto \frac{\Delta G}{G_{0}}\propto e^{d}$$ (4)

However, once most of the sensing area reaches full contact, the current response transitions from a tunneling-dominated regime to one governed by contact mechanics. This stage is better described by Hertzian contact theory, which relates contact area to pressure [23].

$$\frac{∆A}{A_{0}}\propto p^{\frac{2}{3}}$$ (5)

In this regime, the current–pressure response becomes sublinear and gradually saturates as pressure increases. To overcome this issue, we optimized the sensor design by adjusting graphene concentration, dip-coating cycles, fabric layers, and the adhesive pattern.

### Optimization of fabrication for flexible pressure sensing unit

To investigate the optimal piezoresistive performance for flexible pressure sensor, a standard sensing unit was fabricated to evaluate pressure performance. The effective sensing area of this unit was 20 × 20 mm^2^, and a standard pressure was applied using a tensile testing machine, while a digital source meter was used to supply power and measure the current. The sensitivity can be calculated by Eq. 6:

$$\text{Sensitivity}=\frac{∆I/I_{0}}{∆P}$$ (6)

As shown in Fig. 1D, the morphological changes of the polyester cotton substrate were explored under different dip-coating times. Samples were dipped 0 to 5 times in a 2 g/l graphene dispersion, revealing an accumulation of graphene during multiple dipping and drying cycles. It was observed that when fewer dips were applied, the amount of graphene adhered was lower, making it more difficult to form a stable global conductive network. As the number of dips increased, graphene accumulation was evident, indicating that the amount of PVB dissolved in each successive dip coating was significantly lower than the amount of graphene deposited.

Key parameters of the dip-coating process, including graphene concentration and the dip-coated times, were investigated. At low concentrations and with fewer dip-coating cycles, the graphene fabric exhibited poor conductivity and unstable device performance (Fig. S2), making it difficult to form a stable conductive network when encapsulated into the sensing unit. As shown in Table S2, we evaluated sensing unit performance across a 1 to 2.5 g/l graphene concentration with 3 to 6 dip-coated times. Sensing unit prepared with 1 g/l concentration and fewer than 6 dip-coated times exhibited extremely high initial resistance, in some cases beyond the measurement range. Therefore, sensitivity could not be calculated under these conditions. Sensing units coated 3 times with concentrations below 2.5 g/l also showed significant instability due to insufficient surface coverage of graphene. On the other hand, sensing units coated with 2.5 g/l graphene showed poor pressure sensitivity due to excessive surface coverage. Therefore, we focused our detailed sensitivity analysis on sensors fabricated within the 1 to 2.5 g/l concentration range and 3 to 6 dip-coated times. In the high-pressure range, sensitivity showed a trend of initially increasing and then decreasing as the concentration increased, across all dip-coating cycles. This can be attributed to insufficient graphene coverage at lower concentrations, where deformation fails to induce the formation of a conductive network in the affected regions, leading to low sensitivity. At higher concentrations, graphene covered most of the area, resulting in the formation of a pre-existing stable conductive network before deformation. Consequently, the relative increase in the conductive network after deformation was minimal. A moderate graphene concentration (2.0 g/l) was proposed as optimal for covering the polyester cotton without creating an overly dense initial conductive network. At all concentrations, sensitivity decreased as the number of dip cycles increased. This suggests that 4 dip cycles provide sufficient graphene coverage, and additional cycles lead to a too-dense conductive network, reducing the relative change in resistance under compression. As shown in Fig. S3A, the optimal parameters for sensitivity in the low-pressure range were achieved too, which were considered the fabrication parameters for subsequent discussions.

To investigate the changes in fabric surface roughness during the dip-coating process, roughness measurements were performed on the fabric before and after coating. According to the ISO 4287:1997 standard, the Ra and Rz values of the polyester cotton were 5.313 and 17.308 μm, respectively, while those of the graphene fabric increased to 7.883 and 27.382 μm (Fig. S4). This indicates that surface damage occurred after dip-coating, leading to increased roughness. The higher roughness of the graphene fabric facilitates better penetration of the PA into the uneven surface upon melting, resulting in stronger interfacial bonding. X-ray diffraction (XRD) analysis can significantly analyze the deposition of target particles in the substrate [24,25]. XRD measurements of the polyester cotton and graphene fabric were carried out (Fig. S5). The polyester cotton exhibits strong diffraction peaks around 17.5°, 22.6°, and 25°, which can be attributed to the combined contributions of the (110) (~16.4°) and (200) (~22.6°) planes of cellulose I in the cotton, and the (010) (~17.4°), (100) (~22.6°), and (110) (~25.8°) planes of the polyester. The XRD of the graphene fabric remained largely consistent with that of the polyester cotton, indicating that the dip-coating process did not significantly alter the crystalline structure of the polyester cotton. A new peak appears near 27°, which can be preliminarily assigned to the (002) plane of graphene (~26.6°), suggesting that graphene was successfully deposited onto the fiber surface and formed locally ordered structures. This indirectly confirms the effective integration of graphene with the fabric.

**Fig. 2.**
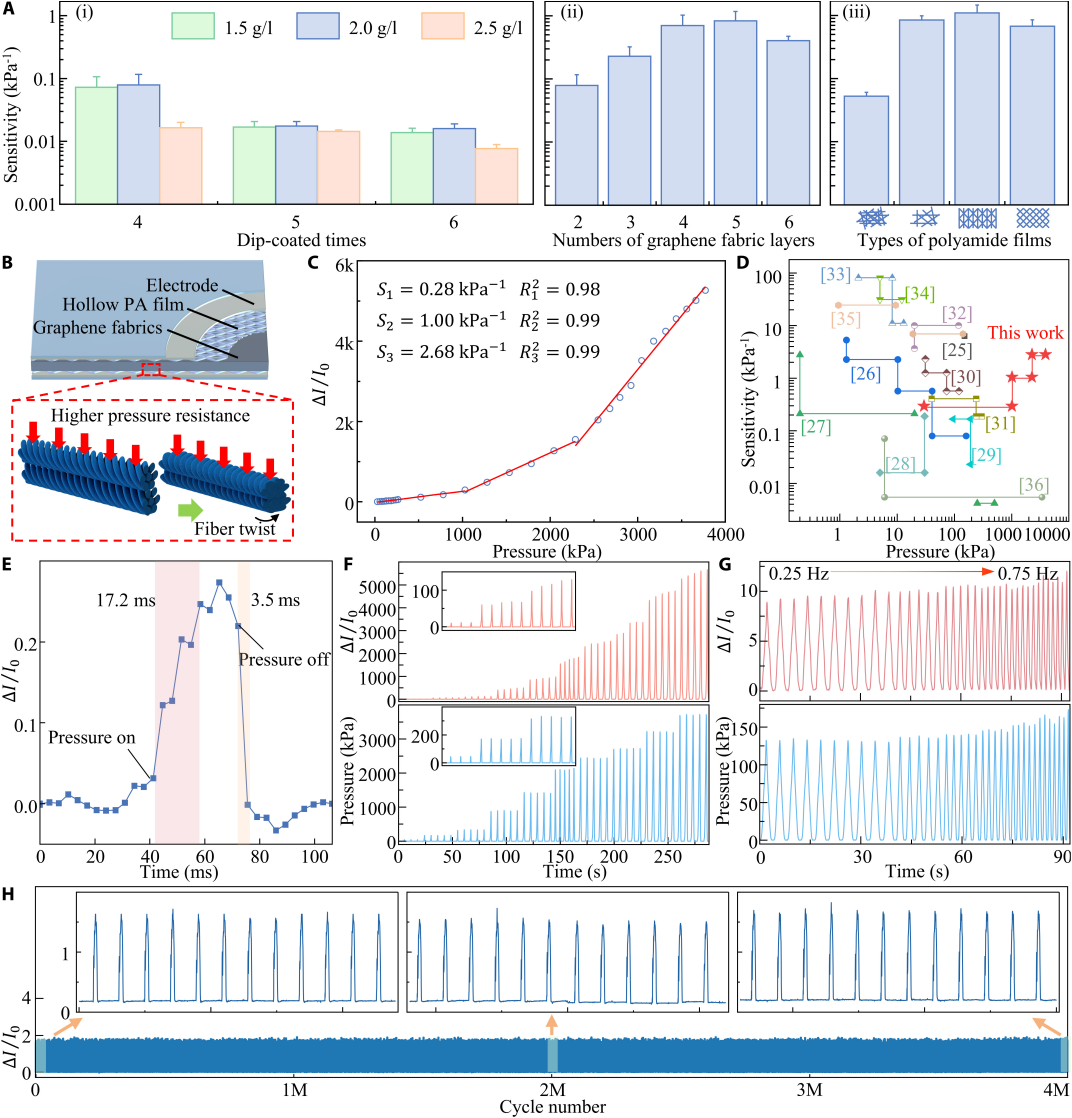
Optimization of the piezoresistive performance of the sensing unit. (A) Influence of various preparation parameters on the performance of the flexible pressure sensing unit: (i) dip-coated times and concentrations; (ii) number of graphene fabric layers; (iii) types of PA films. (B) The designed sensing unit demonstrates an ultra-wide pressure range, attributed to the uniform PA film serving as a stable support and bonding structure, as well as the fiber twisting of polyester cotton fibers under stress. (C) Performance of the flexible pressure sensing unit using the optimal preparation parameters. (D) Comparison of the pressure range and sensitivity with previously reported fabric-based flexible pressure sensors. (E) Response and recovery times of the flexible sensing unit under rapid stimulation. (F) Sensing unit response under different pressure levels. (G) Sensing unit response to different pressure frequency. (H) Stability of the sensing unit over 4 million loading/unloading cycles.

The performance of the sensor was investigated with varying numbers of graphene fabric layers used for encapsulation [Fig. 2A(ii)]. It was found that the sensing unit exhibited optimal performance with 5 encapsulation layers. To investigate the supporting effect and the strength of the introduced tunneling effect of different hollow PA films on the sensing unit, the performance of 4 common types of PA films—23 g/m^2^, 12 g/m^2^, fine grid, and sparse grid (Fig. S6)—was compared. As shown in Fig. S7, the vertical porosities of the 4 types of PA films, calculated through digital image analysis, are 38.5%, 54.7%, 45.0%, and 42.1%, respectively. Compared to other planar patterns, the fine grid films, formed by the superposition of circular grids and stripe patterns, exhibits the greatest thickness (257.4 ± 6.1 μm). As shown in Fig. 2A(iii), the comparison revealed that the fine grid structure yielded the best performance, due to its 3-dimensional (3D) architecture, which facilitates the formation of a deformable support between the graphene fabrics and the electrodes under pressure.

As shown in Fig. 2B, the developed flexible pressure sensor utilizes the PA film as a stable support layer between the electrode and the piezoresistive layer, ensuring robust support and resilience under pressure. Additionally, the piezoresistive layer, composed of twisted polyester cotton fibers, underwent twisting under pressure, thereby dissipating force and withstanding higher pressures. These 2 factors were crucial in enabling the sensor to exhibit an ultra-wide pressure range. The flexible pressure sensing unit, fabricated with the optimal parameter combination, exhibited a sensitivity of 0.28 kPa^−1^ in the pressure range of 29.765 to 1,028.717 kPa, 1.00 kPa^−1^ in the range of 1,028.717 to 2,288.530 kPa, and 2.68 kPa^−1^ in the range of 2,288.530 to 3,770.902 kPa (Fig. 2C). To verify that the sensing unit performance conforms to the superlinear or even exponential relationship indicated by the tunneling effect above, an exponential fit to the optimal device response results was performed as shown in Fig. S8. The fitting equation is shown in Eq. 7, and the fitting coefficient is 0.993, which proves that the superlinear operating range of the sensing unit has covered the needed high voltage range.

$$\frac{∆I}{I_{0}}=496.47\cdot e^{\frac{p}{1,496.3}}-596.02$$ (7)

A performance comparison with existing fabric-based flexible pressure sensors is provided in Fig. 2D and Table S3 [25–36]. Based on this comparison, it is concluded that the developed flexible pressure sensing unit offers an ultra-wide pressure range, addressing the current gap in high-sensitivity flexible pressure sensors for ultra-high pressure ranges, and effectively balances the trade-off between high pressure range and sensitivity.

Figure 2E illustrates the response and recovery times of the developed flexible pressure sensing unit under rapid pressure. When pressure was applied, the sensor reached a steady state within 17.2 ms and recovered within 3.5 ms after the pressure was removed. As shown in Fig. 2F, the stable response of sensing unit under pressure was demonstrated by applying 10 different stepwise pressures ranging from 25 to 3,750 kPa, with each pressure level repeated multiple times. For pressure inputs ranging from 0.25 to 0.75 Hz, overshoot occurred at high-frequency pressure outputs from the tensile testing machine, where the output pressure exceeded the set pressure (Fig. 2G). The sensor effectively captured and replicated this change. To characterize the sensor’s long-term durability, the sensor was subjected to continuous 2.5-Hz load/unload cycles. As shown in Fig. 2H, the sensor’s performance remained stable after 4 million cycles, demonstrating its suitability for daily long-term use.

### Design and testing of flexible pressure sensor array

The main stress-bearing regions of the human plantar include the phalanges, metatarsals, arches, and heels. Different body postures can cause substantial variations in the pressure distribution across these regions. To investigate the influence of human posture on these regions, 5 lower-body models representing different postures were constructed for static finite element simulation. To simplify the simulation, several assumptions were made. First, the gravitational force from the upper body was replaced by a constant force applied evenly across the cut surface of the lower body. Second, the lower body was modeled as a homogeneous material. Finally, the body was assumed to stand steadily on a flat surface, subjected only to gravitational acceleration, with no motion-induced accelerations considered.

**Fig. 3.**
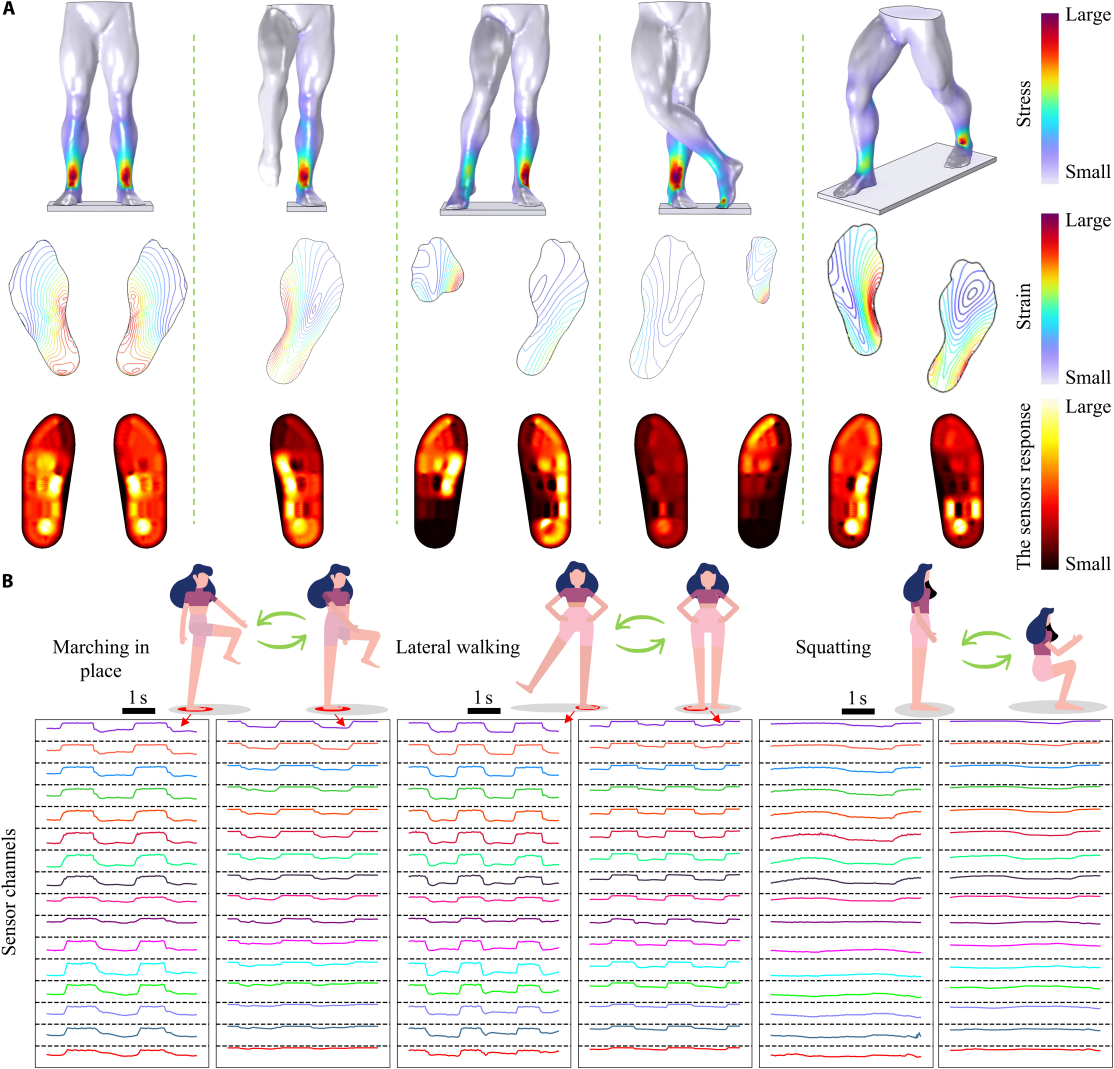
Testing of the flexible pressure sensor array insole. (A) Simulated plantar pressure distributions and sensor array test results under various lower-body postures. (B) Response curves of the flexible pressure sensor array insole during marching in place, lateral walking, and squatting.

As shown in Fig. 3A, 5 postures, including standing, single-leg stance, tap-step stance, cross-leg stance, and forward lunge, were selected for simulation to analyze plantar pressure distribution. Finite element meshing was applied to the established lower-body model (Fig. S9), and the specific simulation settings are provided in Materials and Methods. The simulation results are illustrated in Fig. 3A. The surface of the model exhibited maximum stress near the ankle, which aligned with real-world observations. The simulation results revealed that the areas with the greatest deformation were the lateral heel and arch in different postures. This is because these regions are slightly elevated compared to the touchdown point. Upon landing, they deformed, which prolonged the contact time and alleviated the impact force from the ground. This point was further corroborated by comparing standing and single-leg stance postures, where it was observed that the arch underwent greater deformation as the supporting foot bears more pressure. Although heel and arch do not contact the insole during standing, they deform during certain movements, providing key insights into pressure distribution and body posture.

Reducing the sensor unit size and increasing plantar pressure density improves data completeness. However, it also leads to sensor fragility, complex wiring, higher costs, and slower data sampling. Therefore, the shape and area of the flexible pressure sensing units were designed based on the deformation regions caused by different postures, as revealed by simulation results. To ensure the completeness of information under various postures, areas with consistent deformation were grouped into a unit, minimizing the number of units required. The distribution and shape of the designed units are shown in Fig. S10. The phalanges primarily contribute to maintaining balance and providing forward propulsion, for which 4 sensing units were designed. The metatarsal bones bear most of the pressure, stabilize the midfoot, support the arch, and stabilize the edges, with 6 sensing units placed in this area. The medial and lateral arches absorb shock and support secondary load bearing, respectively. Pressure changes in these regions reflect the body’s lateral movement forces, and 4 sensing units were designed here. The heel, which typically makes contact last during most standard movements, absorbs much of the impact and was designed with 2 units.

The designed shape was laser-cut and encapsulated to form a flexible pressure sensor array in the shape of an insole. The physical picture of the manufactured insole is shown in Fig. S11, and the overall thickness is 0.987 mm. The insole has good flexibility and can still work normally after being bent at a large angle or even folded in half, meeting the actual needs of use. The array’s data acquisition hardware is shown in Fig. S12. The actual plantar pressure distribution was tested under the corresponding postures after wearing the insoles. Additionally, data acquisition tests were conducted under 3 different dynamic postures to collect changes in plantar pressure distribution: marching in place, lateral walking, and squatting. As shown in Fig. 3B and Fig. S13, different sensing units exhibit variations in amplitude or phase during each movement cycle, indicating that each unit can accurately respond to human motion at specific frequencies and capture diverse types of information for further signal processing. Even between similar motions such as marching in place and lateral walking, the sensor array clearly distinguishes differences in periodicity and heel-strike pressure.

To further evaluate the integrity of the developed flexible pressure sensor array for plantar pressure acquisition, it was used to measure pressure distributions under different standing conditions. In medical practice, the duration of maintaining a single leg stand with eyes closed is commonly employed to assess balance ability in the elderly [37]. As shown in Fig. S14A, volunteers performed 5 tasks: double-leg standing, right-leg standing with eyes open, left-leg standing with eyes open, right-leg standing with eyes closed, and left-leg standing with eyes closed. The flexible pressure sensor array successfully captured the rapid adjustments of plantar muscles following balance deviations. During single leg standing with eyes open, the plantar pressure remained stable. However, with eyes closed, oscillations in plantar pressure emerged after a short period, indicating body imbalance and then repeated muscle adjustments to restore equilibrium. The timing of these oscillations partially reflects the volunteers’ balance capacity. Figure S14B presents the mean range and standard deviation of plantar pressure obtained from repeated tests. Regions with larger ranges and variances correspond to the muscles engaged in compensating for imbalance, offering insights into the etiology of poor balance and informing subsequent training strategies.

### Application on pose classification

**Fig. 4.**
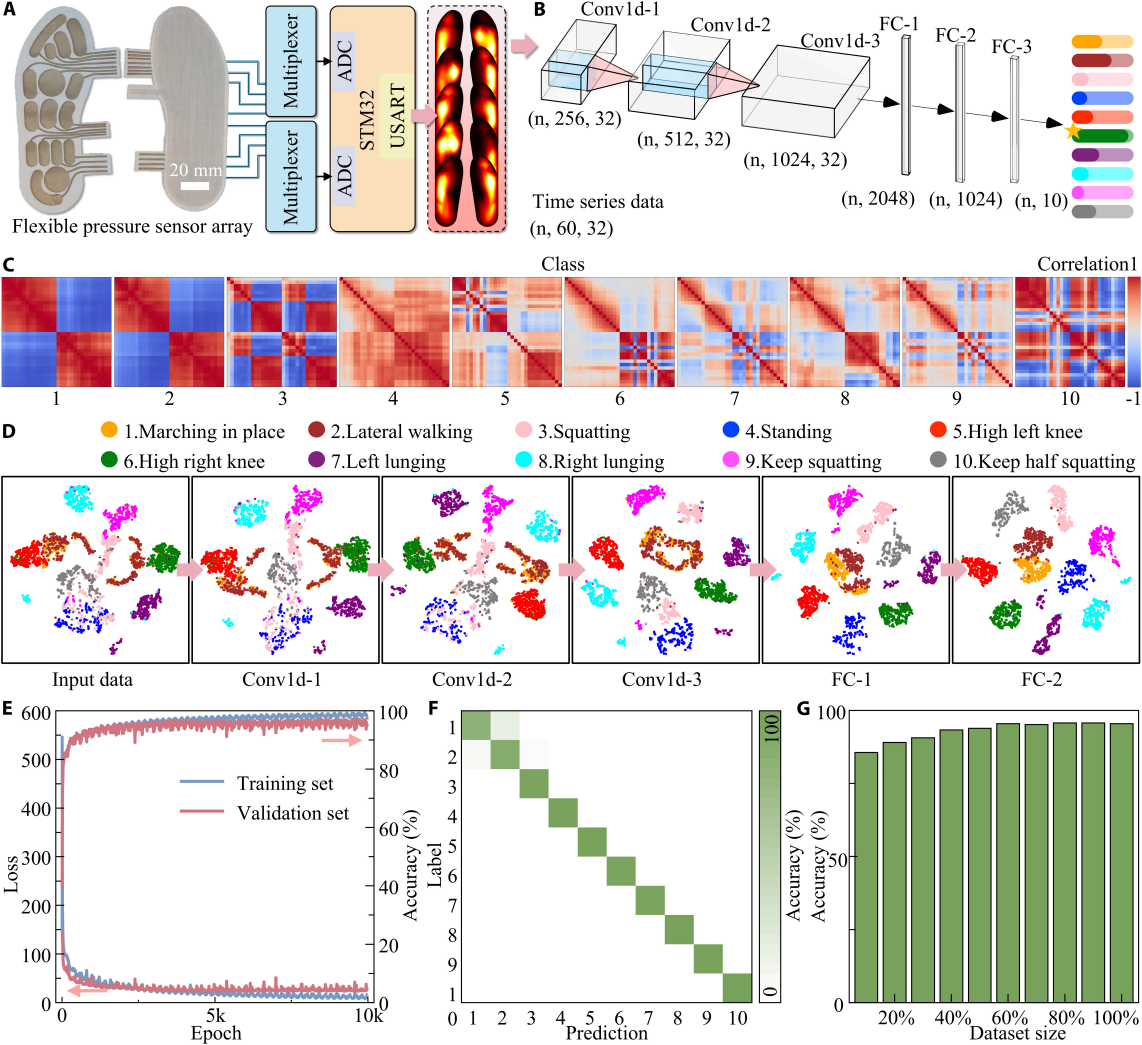
Application of the developed flexible pressure sensor array insole for posture classification tasks. (A) Photograph of the insole and the hardware data acquisition workflow. (B) The developed posture classification model processes time-series signals collected by the insole to classify 10 distinct postures. (C) Heatmap of signal correlations under 10 different postures based on the sensor array data. (D) *T*-distributed stochastic neighbor embedding dimensionality reduction analysis of input data and intermediate features processed by each layer of the posture classification model. (E) Loss and classification accuracy curves for the training and validation sets during model training. (F) Confusion matrix of the predictions made by the trained posture classification model on the test set. (G) Prediction accuracy of the posture classification model on the test set as the increasing training data increases.

To demonstrate that the developed insole-shaped flexible pressure sensor array can collect comprehensive plantar pressure information, a 10-posture classification task was conducted. Among the 10 postures, 3 were dynamic and 7 were static, including marching in place, lateral walking, squatting, standing, high left knee, high right knee, left lunging, right lunging, keep squatting, and keep half squatting. Sensor signals were collected from volunteers while they maintained these postures, and actions were classified based solely on these signals, with further details provided in Materials and Methods. As shown in Fig. 4A, data from the 2 flexible pressure sensor arrays were transmitted through multiplexers and sampling circuit to the 2 analog-to-digital converters (ADCs) of the STM32. After analog-to-digital conversion, the data were packaged in a predefined protocol and transferred to a personal computer via universal synchronous asynchronous receiver transmitter (USART) for storage. As shown in Fig. 4B, 1-s data are input into the developed pose classification model to classify the different postures.

As shown in Fig. 4C, correlation analysis was conducted on each sensing channel under different postures. In marching in place and lateral walking, the sensing units of the left and right feet showed a strong negative correlation, while those within each foot exhibited a high positive correlation. This was due to the pressure changes caused by alternating leg movements during walking. In the squatting posture, the first few sensors corresponding to the phalanges and metatarsals showed high correlation, which is because the volunteer kept the heel elevated to balance body. All sensing units showed positive correlation in the standing posture, consistent with the simultaneous pressure applied. In the left and right high knee postures, the correlation between the weight-bearing foot was consistent with the pressure in standing, while pressure on the sole of the elevated foot was random. The varying correlations between sensing units in different postures were key to the model’s ability to accurately classify them.

To explore the process through which the model categorizes the input data into 10 classes, *t*-distributed stochastic neighbor embedding (t-SNE) dimensionality reduction was applied to the input data and the outputs of each layer (Fig. 4D). In the input data, the 10 classes were mixed, with no clear decision boundaries. As the model processed the data layer by layer, the classes were gradually separated. The hardest postures to distinguish were marching in place and lateral walking. They were first separated at the FC-1 layer and more clearly at FC-2. This difficulty arose because slow or changing-direction lateral walking resembled marching in place. However, lateral acceleration and force during side-to-side movement allowed them to be differentiated.

As shown in Fig. 4E, during the model training, the loss of the training and validation sets gradually decreased until approximately 2,500 batches, after which overfitting occurred, and the validation loss plateaued. A similar trend is observed in the classification accuracy of the training and validation sets. The oscillations observed are due to the use of a cosine annealing learning rate schedule, which adjusted the learning rate during training to prevent convergence to a local optimum. The results of the optimal model on the test set are shown in Fig. 4F, with an accuracy of 95.5%. The confusion matrix for the test set demonstrates the classification accuracy across different categories, with the exception of a small number of misclassifications between marching in place and lateral walking, which also confirms the classification performance shown in Fig. 4D. Figure 4G shows that the accuracy of the test set increases with the amount of training data, indicating that the model can handle more complex tasks by incorporating additional data.

### Application on pose estimation

**Fig. 5.**
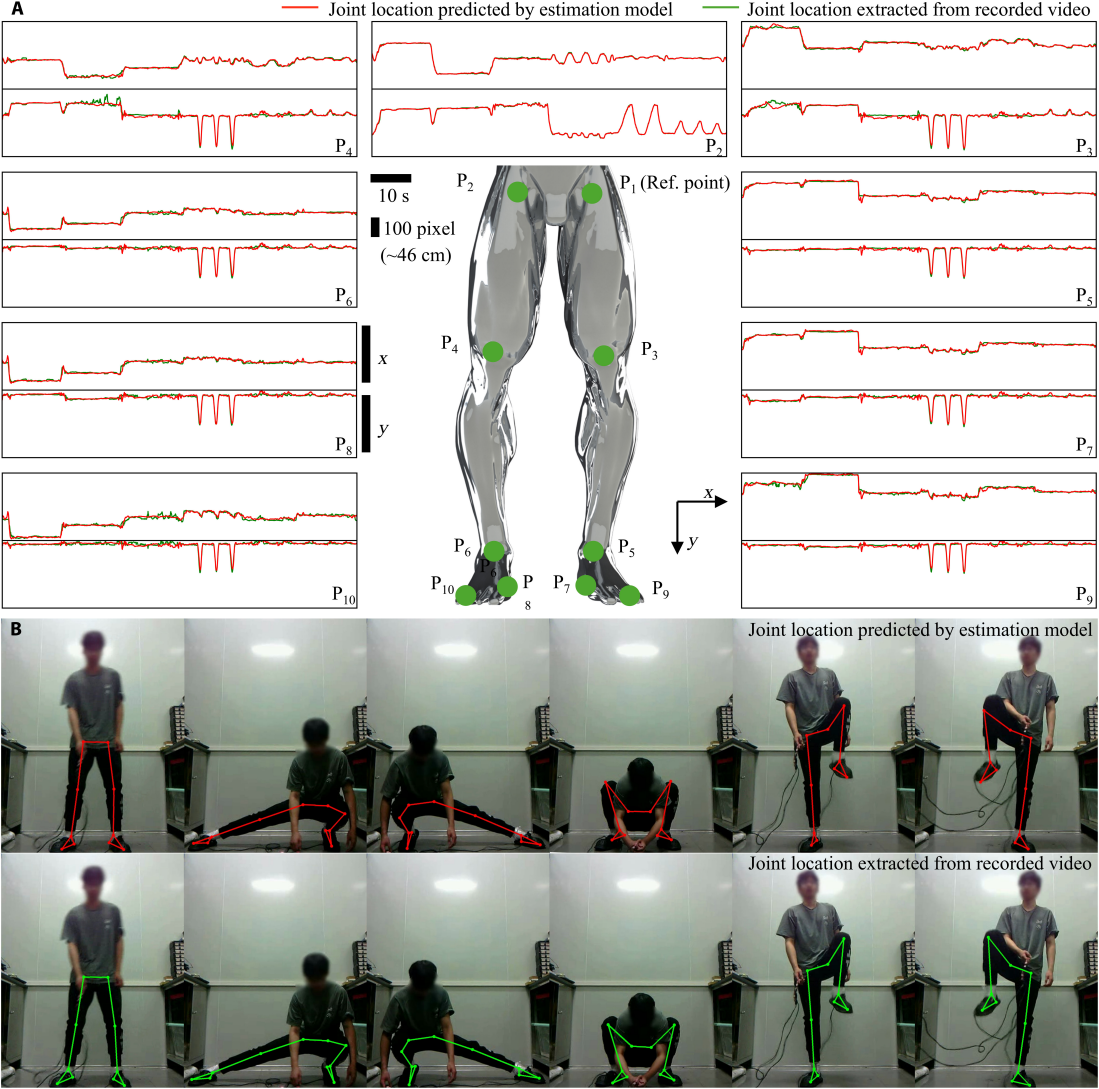
Application of the developed flexible pressure sensor array insole for pose estimation tasks. (A) Schematic representation of predicted lower limb joints, with a comparison between predicted locations and ground truth labels for each point. (B) Comparative test results showcasing the model-predicted joint positions with joint positions extracted from recorded video.

After demonstrating that the flexible pressure sensor array can capture sufficient data to distinguish between different postures, its performance in pose estimation was evaluated. As shown in Fig. 5A, 10 key points, including hip, knee, ankle, heel, and toe on both the left and right legs, were selected as targets. Data acquisition involved simultaneous video capture of human activity using a camera, along with synchronous recording of the output signals from the worn flexible pressure sensor array. Joint locations (labels) were extracted from each video frame using a mature and accurate visual human pose estimation model. The time stamps for each video frame and each sensor data frame were recorded to ensure proper alignment, as shown in Fig. S15. Due to the combined influence of gravitational and kinematic accelerations during human motion, static and dynamic actions, even at the same position, can result in significantly different plantar pressure distributions. This issue was addressed by incorporating data from a longer temporal window. Specifically, sensor data from the previous 2 s were used as input, and the data were encoded and features were extracted by a transformer encoder.

A transformer-based time series regression model was proposed to efficiently tackle the pose estimation task in time series (Fig. 1A). This model was designed to predict joint locations without generating sequences, utilizing only the encoder component of the transformer to leverage its powerful feature extraction capabilities. The encoder captures global dependencies between different time steps through a multi-head self-attention mechanism, extracting discriminative features. The encoder’s output was down-sampled via global average pooling along the time dimension to eliminate redundant information, and the final regression output was produced by a fully connected network. Compared to traditional convolutional neural networks and recurrent neural networks, the proposed model captures long-range dependencies more efficiently and is simpler and more computationally efficient. It reduces computational cost while maintaining high performance, showing strong generalizability and practical potential.

Figure 5A shows the temporal changes in the coordinates of each joint during a movement. Since joint locations were extracted from a 2D image, the depth dimension was not considered (Fig. S16). The flexible pressure sensor can capture posture data but does not provide the human location in the image. Therefore, the other joints were referenced relative to P1 to obtain relative coordinates for model prediction. The model-predicted joint locations closely match those extracted from the image, with an average error of 7.8 pixels (~3.6 cm) across the test set. A clearer comparison of the prediction results is provided in Fig. 5B and Movie S1, where the volunteer was wearing the flexible pressure sensor array, and the developed pose estimation model accurately predicted the joint locations.

**Fig. 6.**
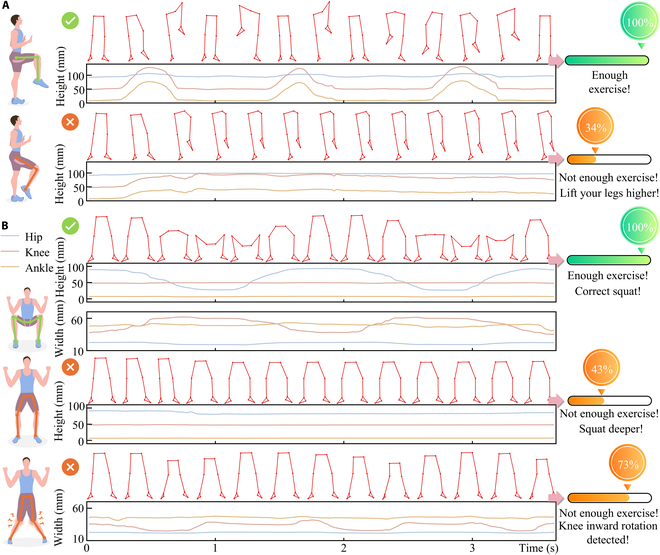
Application of the developed lower limb motion capture system in rehabilitation exercise assessment. (A) Detection of movement during leg-raising rehabilitation exercises. (B) Evaluation of movement adequacy and correctness during squatting rehabilitation exercises.

To validate the practical applicability of the developed lower limb motion capture system, a simulated monitoring of the rehabilitation process was conducted for further investigation. For patients with weakened or injured lower limbs, rehabilitation through simple leg-raising and squatting exercises is a common approach. However, current methods lack remote monitoring capabilities for clinicians or patients to assess the sufficiency and accuracy of rehabilitation exercises. As shown in Fig. 6A, after wearing the developed insoles, the volunteer performed normal leg-raising movements and simulations of insufficient leg-raising height. The developed lower limb motion capture system utilized the flexible pressure sensor array insoles to collect plantar pressure distribution data during movement and predict the joint status of the lower limb. The movement adequacy (MA) was quantified by the ratio of the lifted thigh angle to the target angle (Fig. S17). Here, MA was calculated by Eq. 8:

$$\mathrm{MA}=\max\left(\frac{\text{arccos}\left(\frac{h_{\mathrm{hip}1}-h_{\text{knee}1}}{h_{\mathrm{hip}0}-h_{\text{knee}0}}\right)}{\theta_{\text{target}}}\right)$$ (8)

where $h_{\mathrm{hip}0}$, $h_{\text{knee}0}$, $h_{\mathrm{hip}1}$, $h_{\text{knee}1}$, and $\theta_{\text{target}}$ express hip height in standing, knee height in standing, hip height in motion, hip height in motion, and target angle, respectively. The target angle used for the evaluation here was 120°.

In the simulated insufficient leg lift exercise (Fig. 6A), the volunteer imitated a rehabilitation patient with limited leg strength attempting to reach the target height. The system detected an MA of 34% and indicated the need for a higher leg lift to complete the rehabilitation exercise. Figure 6B illustrates insufficient movement and incorrect squatting during the squatting process. First, the volunteer performed a correct squat, lowering the hips to achieve the target thigh flexion angle, and the system confirmed a correct squatting posture and sufficient movement. Subsequently, the volunteer simulated a patient with insufficient leg strength, struggling to complete a full squat. The system detected an MA of only 43% and suggested a deeper squat. Furthermore, the volunteer simulated a common knee inward rotation error during squatting in rehabilitation exercises. The system detected an MA of 73% while simultaneously measuring the distances between the knees and ankles, identifying an inward knee rotation. The system then provided feedback to correct the squatting posture to enhance rehabilitation effectiveness and prevent potential joint injuries.

## Discussion

In summary, flexible pressure sensor arrays were utilized to develop a novel approach for estimating human lower limb posture. The developed sensing unit was fabricated through dip-coating, laser layered selective direct writing, and hot-press encapsulation. By introducing a PA layer to enhance the tunneling effect between the electrode and the piezoresistive layer, the sensing unit achieved a pressure range of 3,770.9 kPa and a sensitivity of 2.68 kPa^−1^. These capabilities enable accurate capture of peak plantar pressure signals encountered in daily activities. Compared to reported fabric-based flexible pressure sensors, the proposed sensors offer high sensitivity and a significantly wide pressure range. The response and recovery times of 17.2 and 3.5 ms, respectively, ensure rapid response to dynamic pressure changes, while the sensor’s durability exceeds 4 million cycles, meeting daily insole usage requirements.

The fabricated sensor array was employed for lower limb pose classification and estimation tasks. In the classification task, including 10 static and dynamic postures, the system achieved a high classification accuracy of 95.5% using sensor-captured signals. After confirming the array’s capability to acquire sufficient data for accurate classification, the pose estimation task was evaluated. During various movements, the sensor array recorded plantar pressure distributions and estimated joint poses with a prediction error of approximately 7.8 pixels (~3.6 cm), demonstrating the method’s accuracy for lower limb pose estimation.

This system only requires users to wear insoles embedded with the flexible sensor array, ensuring minimal interference with natural activities and excellent comfort. Its seamless integration into daily life can make users virtually unaware of its presence, positioning it as an ideal motion capture solution. Currently, the system holds significant expectation in applications such as film production, virtual avatars, virtual reality/augmented reality (VR/AR) gaming, sports, and medical rehabilitation. In the long term, it could serve as a comfortable, natural, and indispensable data input interface for every next-generation Internet user.

## Materials and Methods

### Preparation of graphene fabrics

To prepare graphene/PVB/ethanol dispersion, 1 g of PVB (98 wt %, Sigma-Aldrich) was dissolved in 1 l of ethanol. Next, 2 g of monolayer graphene (Shenzhen Jiasheng New Material Co.) was added to it and dispersed using ultrasonic agitation to yield a graphene/PVB/ethanol dispersion. Then, polyester cotton (Dongguan Huamei buyi Co. Ltd.) with specific shapes was dipped into the dispersion and placed onto a Cu-plated phenolic resin plate for drying at 60 °C for 10 min. This dipping and drying process was repeated 4 times to fabricate the graphene fabrics.

### Laser direct writing on flexible substrate

The electrode array and common ground electrode were fabricated using a selective laser direct writing. First, Cu–Ni plated fabric (Chenxi Technology Electronic Application Co. Ltd.) with an adhesive backing was affixed to polyester cotton. Then, the Cu–Ni plated fabric was cut with an infrared laser machine (Han’s Laser Technology Industry Group Co. Ltd., 20 kHz, 1,064 nm) at low power processing parameters (17.5%, 50 mm/s), preventing damage to the polyester cotton. After the electrode pattern was cut, both the Cu–Ni coated fabric and polyester cotton were simultaneously cut using high-power laser processing parameters (70%, 10 mm/s) to obtain the desired electrode array and common ground electrode.

### Assembly of the flexible pressure sensor array

After arranging the electrode array, hollow PA film (Jiaxing Jiajiahe Hot Melt Adhesive Co.), graphene fabric, hollow PA film, and common ground electrode, the flexible pressure sensor arrays were assembled by heat pressing using a hot stamping machine (Yiwu Kuti Digital Imaging Co.) at 170 °C for 45 s. The 4 PA films—23 g/m^2^, 12 g/m^2^, fine grid, and sparse grid—have measured thicknesses of 166.0 ± 2.3 μm, 139.8 ± 2.1 μm, 257.4 ± 6.1 μm, and 133.8 ± 2.1 μm, respectively. Their actual basis weights are 21.2 ± 0.3 g/m^2^, 13.5 ± 0.6 g/m^2^, 26.1 ± 0.5 g/m^2^, and 30.2 ± 0.4 g/m^2^, respectively. Using the same encapsulation method, the flexible pressure sensing unit used to investigate the piezoresistive performance was prepared with 40 × 40 mm^2^ polyester cotton, 40 × 40 mm^2^ hollow PA film, and 20 × 20 mm^2^ graphene fabric.

### Characterization of graphene fabric

The surface roughness measurements were conducted using an optical 3D surface profilometer (Super View W1, Chotest Technology Inc.) in accordance with the ISO 4287:1997 standard. XRD analysis was conducted using a Cu Kα radiation source (λ₁ = 1.5406 Å, λ₂ = 1.5444 Å, Kα₂/Kα₁ intensity ratio = 0.5) at 40 kV and 40 mA. Scans were performed over a 2θ range of 10° to 50° with a step size of 0.013°. The vertical resistivity of the graphene fabric was measured by applying 125 kPa to a single layer of fabric and recording the resistance between its top and bottom surfaces.

### Setting of finite element simulation

The lower limb models, as shown in Fig. 3A, was imported into the finite element simulation software. A relative simplification tolerance of 0.01 and a defect removal factor of 1 were then applied to simplify the model and reduce surface complexity. The material of the plate was set to rubber, and the human body was modeled as a homogeneous material with skin properties, having a density of 1,109 kg/m^3^, Young’s modulus of 14 MPa, and Poisson’s ratio of 0.4. A downward vertical force of 300 N was applied to the upper section of the lower limb, simulating the pressure from the upper body. Fixed constraints were applied to the bottom surface of the plate to simulate the force experienced when standing on rubber. A gravity term was added to the model, with gravitational acceleration set to 9.8 m/s^2^ in the downward direction. Free tetrahedral meshing was performed on the model, as shown in Fig. S9. After performing the steady-state pressure finite element simulation, the stress distribution on the human body surface and the deformation contour map of the foot sole were exported for comparison with the actual pressure distribution.

### Collection of datasets

The pose classification dataset was collected from volunteers wearing 2 flexible pressure sensor arrays shaped like insoles while performing specified poses. The insole-shaped flexible pressure sensors, shown in Fig. 4A, contain a total of 32 sensing units. The data from these sensing units were acquired using a developed resistive scanning array (Fig. S12A). These sensors were connected to a common ground, while the other terminals were linked to a 4-to-16 multiplexer. Sequential readings of 16 data points were achieved by controlling 4 sets of logic signals. Due to unavoidable voltage drop during signal transmission through the multiplexer, calibration of the digital values and resistance was performed, as shown in Fig. S12B. The reading speed of the sensor array was approximately 30 sps.

The pose estimation dataset was collected from volunteers wearing the sensor arrays while performing a series of movements. The joint positions of the volunteers were extracted from images captured by a camera using OpenCV. The video was sampled at 15.5 samples per second (sps), and data alignment was achieved by recording the timestamp of the serial port data and the timestamp of the video. Since extracting depth-related positional information from images is inherently less accurate, the volunteers were instructed to perform activities within the same depth plane whenever possible. Pixel measurements of the volunteers’ hip widths were converted into physical units using a calibration factor based on the pixel-to-physical ratio (Fig. S16).

### Model training

The pose classification dataset was segmented using a nonoverlapping 18-frame time window applied to the whole serial data. To avoid introducing inaccurate pose data, a 2-s window during transitions between different poses was excluded. A total of 5,555 data samples were obtained and randomly split into training, validation, and test sets in an 8:1:1 ratio. The pose classification model, built using PyTorch, is illustrated in Fig. 4B, and training was conducted using the designated training set. The model was trained for 10,000 epochs with a batch size of 256, a learning rate of 10^−4^, and a random seed set to 0. Cross-entropy loss was employed as the loss function, and parameters were optimized using the Adam optimizer with a weight decay of 10^−3^. A cosine annealing learning rate scheduler with a period of 100 and a minimum learning rate of 10^−6^ was applied to dynamically adjust the learning rate during training. The detailed architecture and parameters of the model are presented in Table S4.

The pose estimation dataset was created by segmenting the complete matched data into 60 equal-length segments, which were then divided into training and test sets in a 9:1 ratio. The video data were segmented with a step size of 1 frame and a length of 30 frames. The pose in the final frame of each segment was used as the label, while the serial data corresponding to the 30-frame time window served as the model input. The total dataset consisted of 81,256 samples, with 8,121 samples allocated to the test set. A transformer-based model was trained using 5-fold cross-validation. The training process involved 1,000 epochs with a batch size of 256, a learning rate of 10^−3^, and a random seed set to 0. Parameter optimization was performed using the Adam optimizer with a weight decay of 10^−5^. During training, a cosine annealing learning rate scheduler was employed with a period of 30 and a minimum learning rate of 10^−5^ to dynamically adjust the learning rate. A composite loss function combining L1 and L2 losses was utilized, with weights of 1 and 10 assigned to L1 and L2, respectively. To prevent gradient explosions during training, gradient clipping was applied. The detailed parameters of the model are provided in Table S5.

## Data Availability

All data are available in the main text or the Supplementary Materials.
