# Supplementary material for: Fabric-Based Flexible Pressure Sensor Arrays with Ultra-Wide Pressure Range for Lower Limb Motion Capture System
Source: Research (Wash D C). 2025 Aug 18;8:0835. doi: 10.34133/research.0835 (PMC12358749; doi:10.34133/research.0835)
Supplement: Supplementary 1 — Figs. S1 to S17 Tables S1 to S5 Movie S1 [file research.0835.f1.zip › R2-SupplementalMaterial.pdf]

Research
A SCIENCE PARTNER JOURNAL
SPJ
SCIENCE PARTNER JOURNALS

Supplemental Material

Title

Fabric-Based Flexible Pressure Sensor Arrays with Ultra-Wide Pressure Range for Lower Limb Motion Capture System.

Authors

Xiaohua Wu[1], Yuxuan Liang[1], Longsheng Lu[1], Shu Yang[1], Zhanbo Liang[1], Feilong Liu[1], Xiaoyu Lu[1], Bowen Xiao[1], Yilin Zhong[1], Yingxi Xie[1*]

Affiliations

Xiaohua Wu[1], Yuxuan Liang[1], Longsheng Lu[1], Shu Yang[1], Zhanbo Liang[1], Feilong Liu[1], Xiaoyu Lu[1], Bowen Xiao[1], Yilin Zhong[1], Yingxi Xie[1*]

[1]School of Mechanical & Automotive Engineering, South China University of Technology, Guangzhou 510641, China.

[*]Address correspondence to: xieyingxi@scut.edu.cn

SUPPLEMENTARY MATERIALS

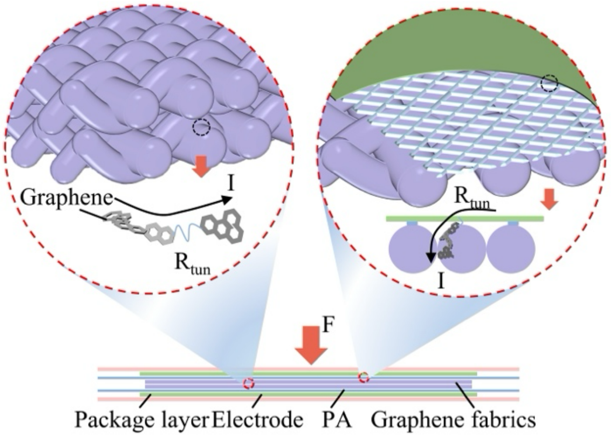

Fig. S1. Tunneling effect of sensor during compression.

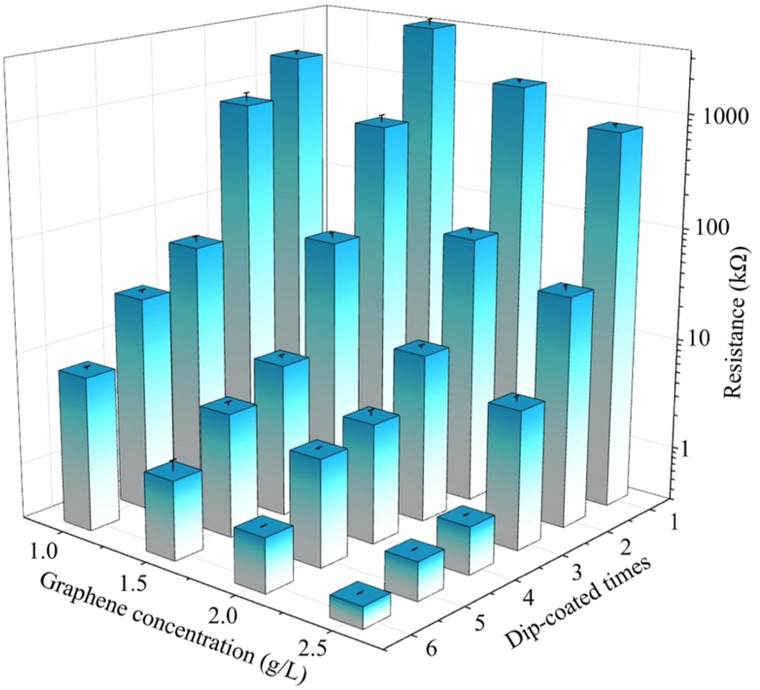

Fig. S2. Resistance under 125 kPa pressure of graphene fabrics prepared by dip coating concentration and number of times.

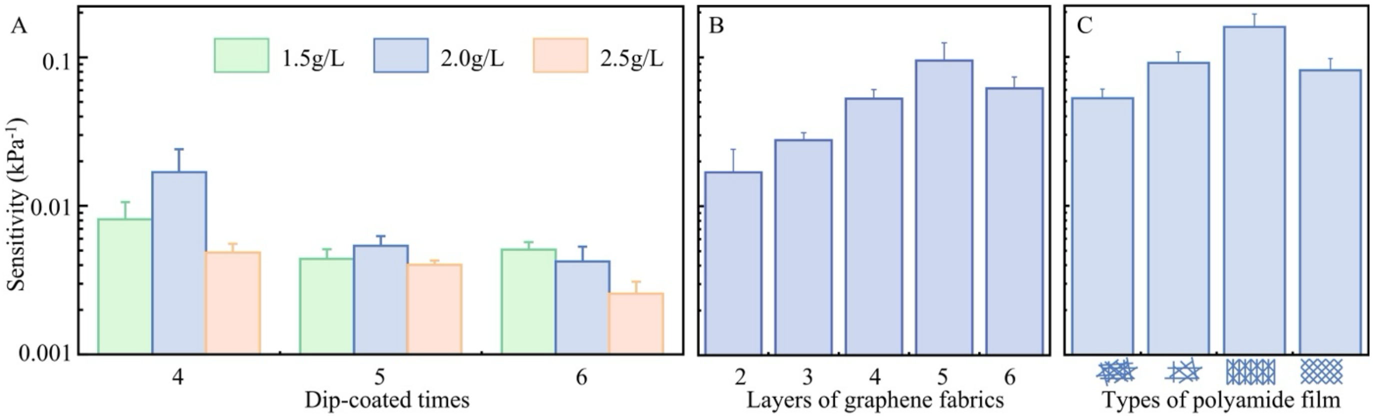

Fig. S3. Various preparation parameters on the performance in low pressure range of the flexible pressure sensing unit. (A) Dip-coating cycles and concentrations. (B) Number of graphene fabric layers. (C) Types of PA films.

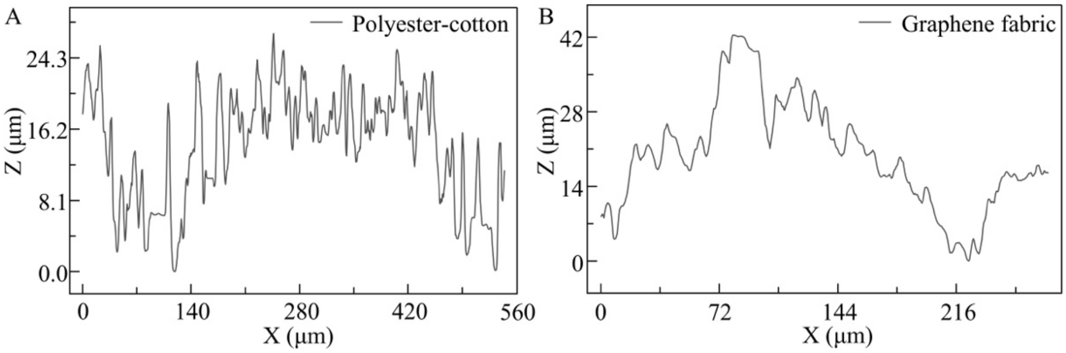

Fig. S4. Optical measurement results of roughness of polyester-cotton (A) and graphene fabric (B).

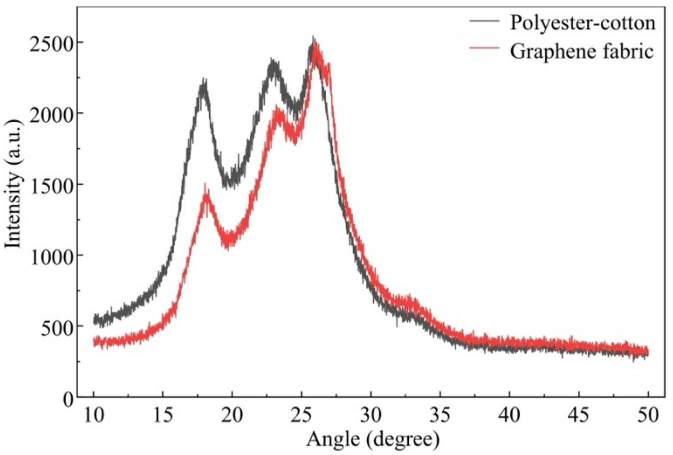

Fig. S5. XRD of polyester-cotton and graphene fabric.

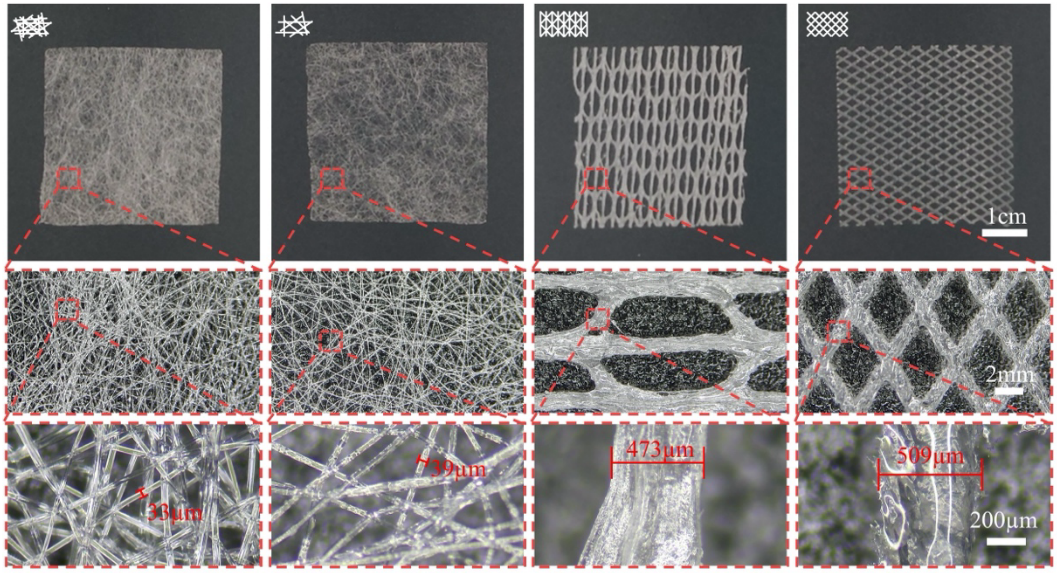

Fig. S6. Physical images and micro-nano structural images of four types of PA films.

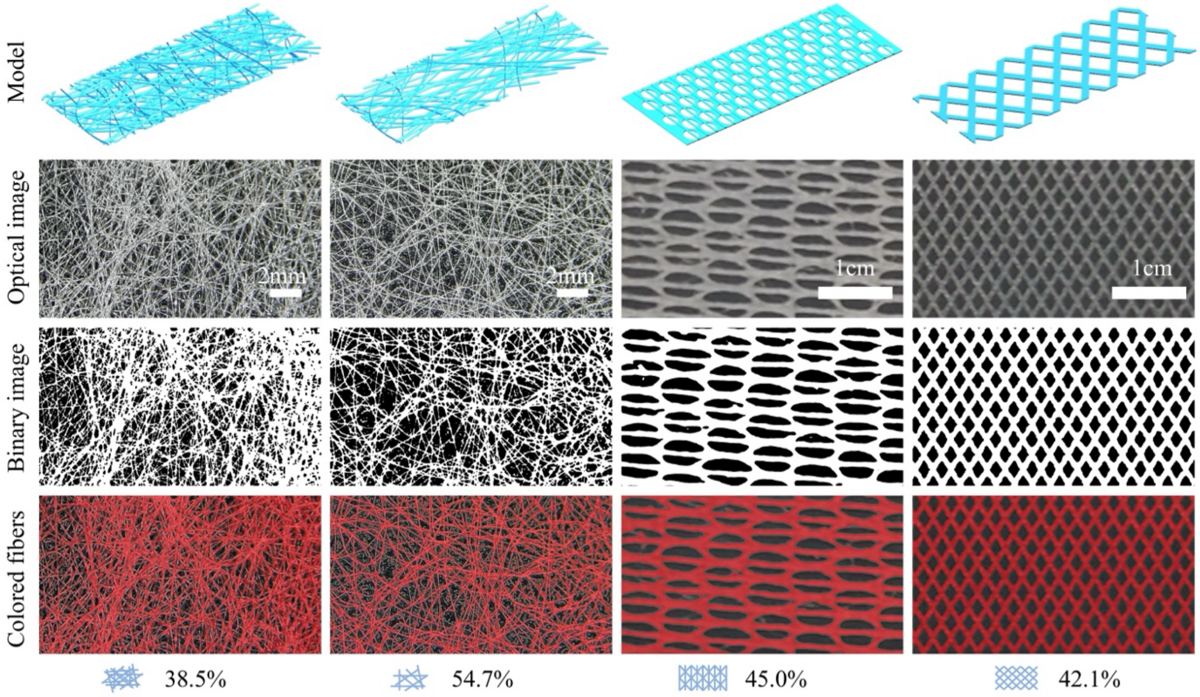

Fig. S7. Model and porosity of PA films.

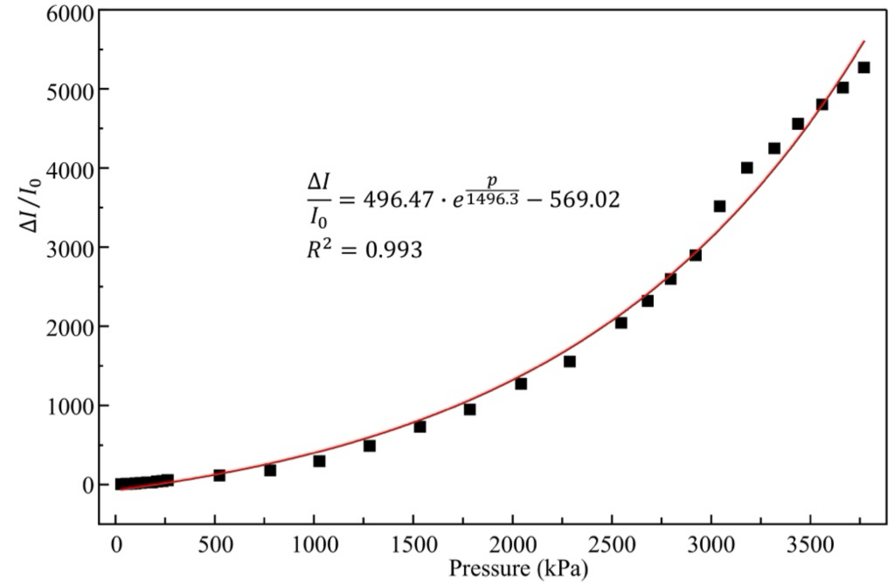

Fig. S8. Sensing unit performance and fitting relationship for optimal parametric fabrication.

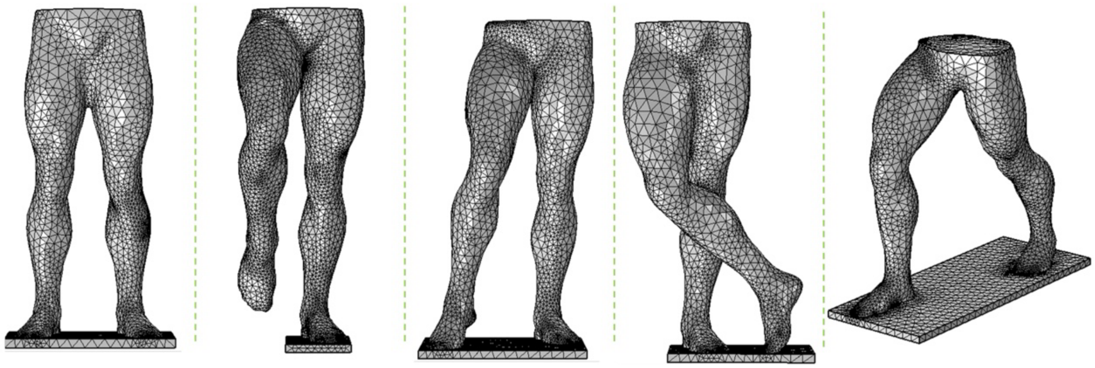

Fig. S9. The tetrahedral mesh effect of five different lower limb postures in finite element simulation.

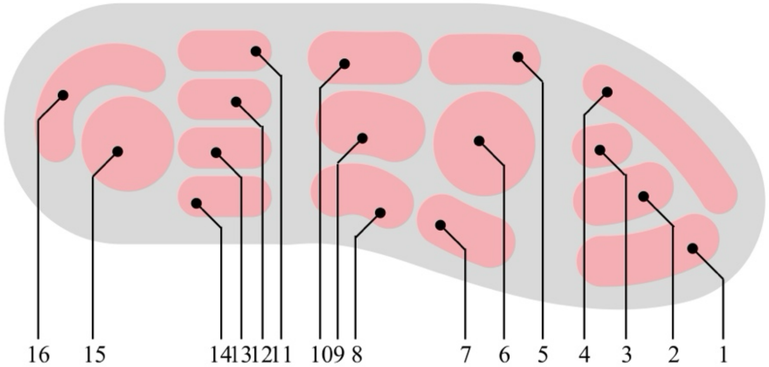

Fig. S10. The designed shape and arrangement of the insole electrodes.

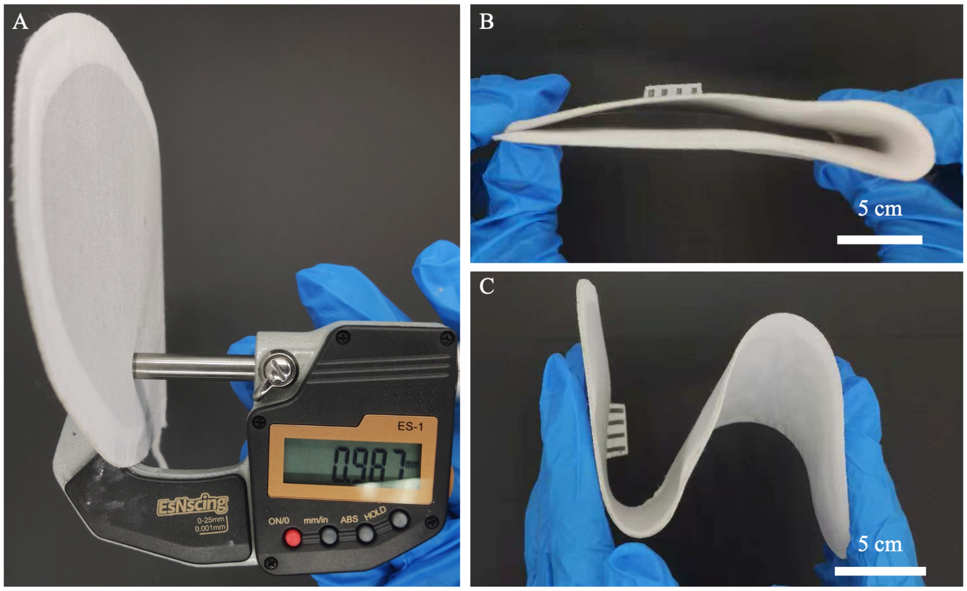

Fig. S11. Photograph of the developed flexible pressure sensor array. (A) Thickness of the sensor array; (B) Sensor array under folding; (C) Sensor array under bending.

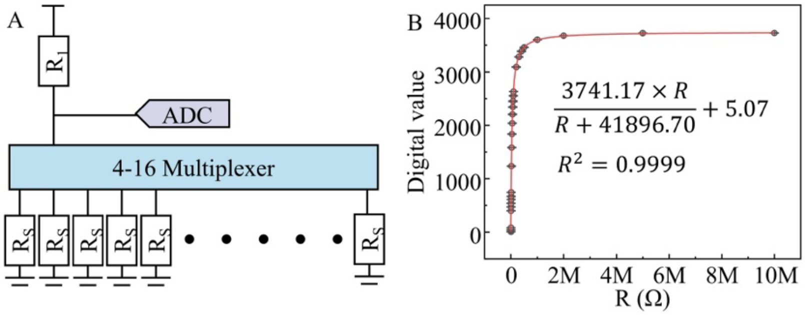

Fig. S12. The developed data acquisition system for the flexible pressure sensor array. (A) A schematic diagram of the connection between the sampling circuit and the multiplexer. (B) The calibration results of the hardware circuit after taking into account the internal resistance of the multiplexer.

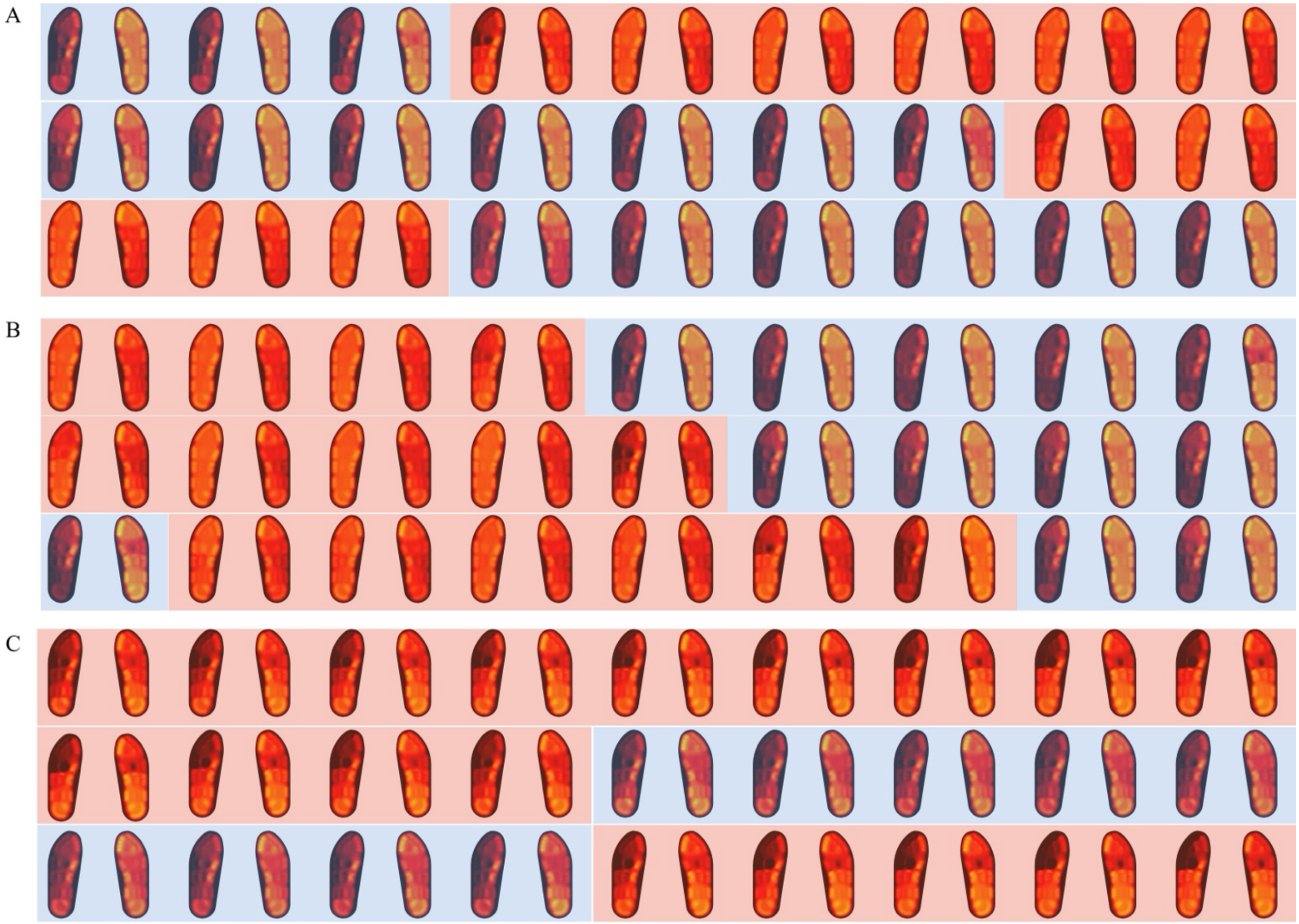

Fig. S13. Plantar cloud images during marching in place, lateral walking and squatting.

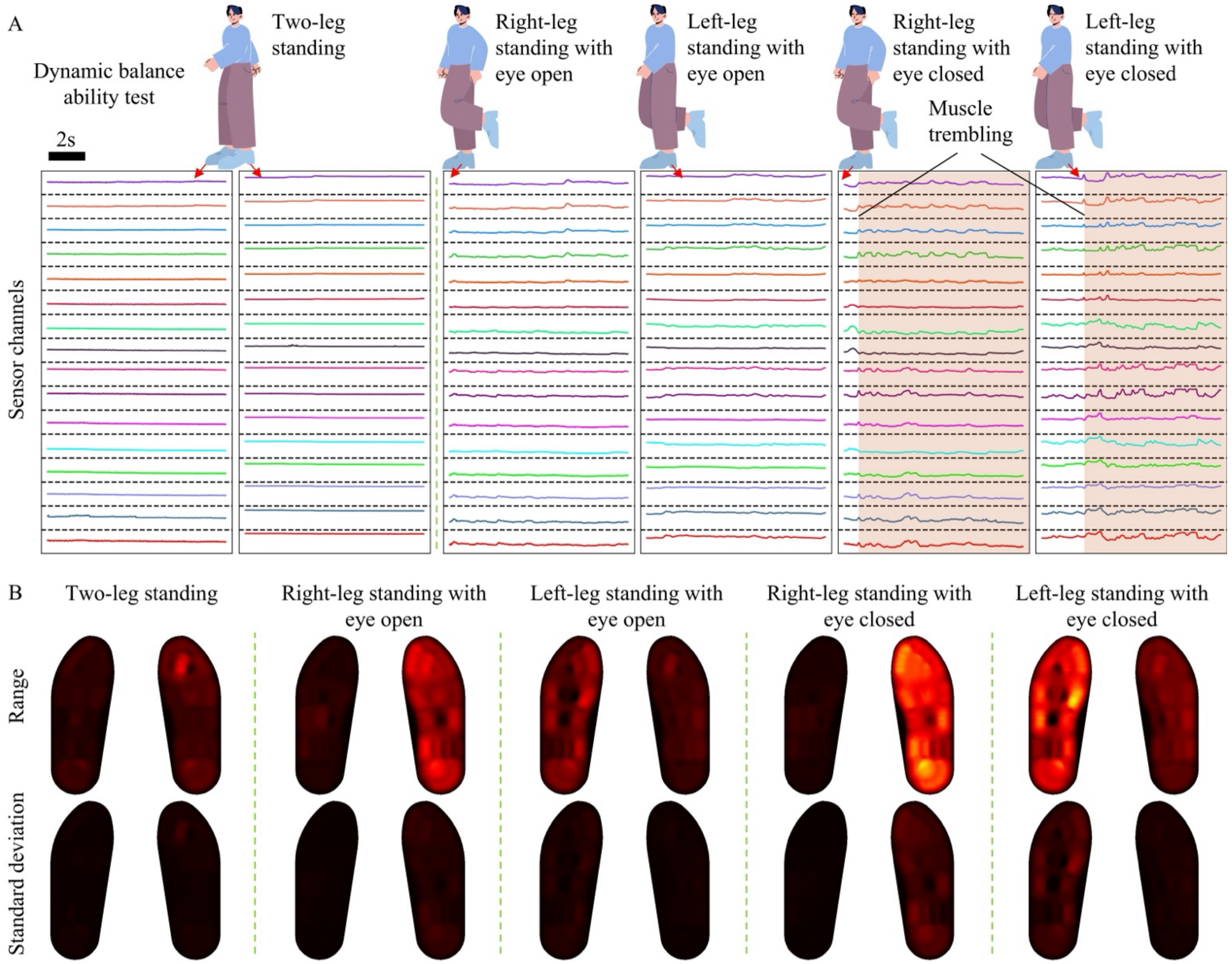

Fig. S14. Application of the developed flexible pressure sensor array in assessing human balance during standing tests. (A) Plantar pressure signal acquisition under various standing conditions. (B) Mean range and standard deviation of plantar pressure recorded across repeated trials of standing postures.

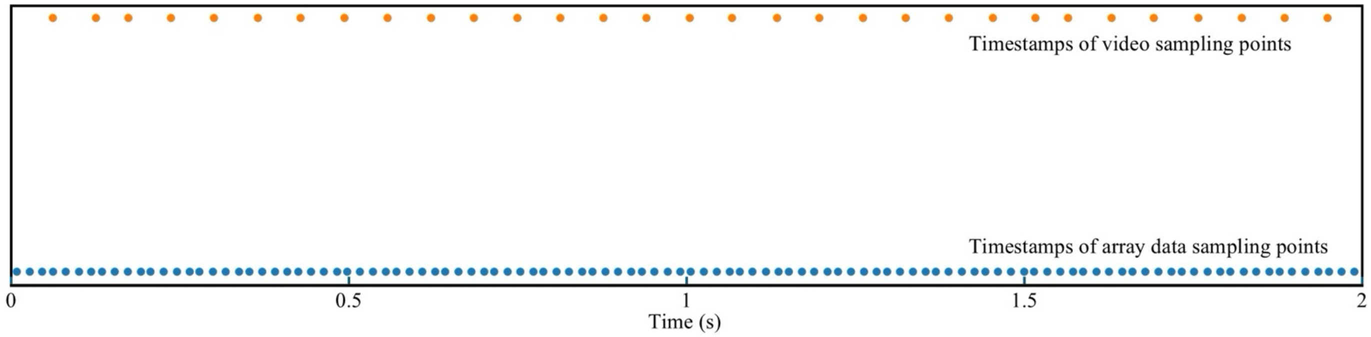

Fig. S15. Comparison of video sampling points and foot sole pressure distribution sampling points in the pose estimation dataset.

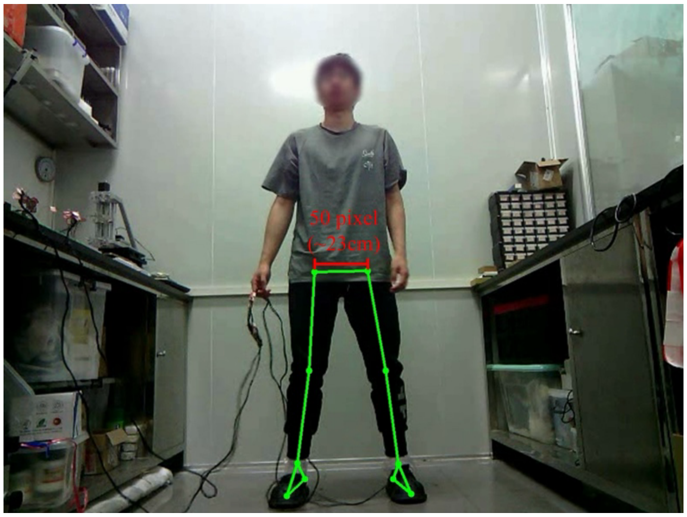

Fig. S16. Calibration of pixel length using hip bone width measurements.

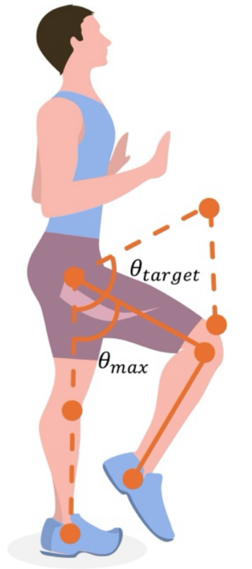

Fig. S17. The movement adequacy is quantified by calculating the maximum and target angles of thigh lift.

Table S1. Comparison of the performance of the developed flexible pressure sensor unit with reported fabric-based flexible pressure sensors.

Sensor type	Wearing position	Product form	Function	Ref.
Optical cameras	Environmental space installation	-	Human posture reconstruction	[7]
Inertial sensors	Arms, legs, head, chest	Ten adhesive devices	Human posture reconstruction	[8]
Strain sensors	Full body joints	Sensing suit	Human posture reconstruction	[17]
Strain sensors	Back waist, shoulders, elbows, knees	Sensing suit	Human posture reconstruction	[18]
Pressure sensors	Calf	Insole	Finite postures classification	[4]
Pressure sensors	Sole	Insole	Finite postures classification	[20]
Pressure sensors	Sole	Insole	Human posture reconstruction	This work

Table S2. Sensing unit sensitivity prepared with different dip coating parameters

Dip-coated times	Graphene concentration	High pressure sensitivity	Standard error
3	1.5	0.149480	0.094066
3	2	0.353688	0.151307
3	2.5	0.026064	0.002237
4	1.5	0.072458	0.034893
4	2	0.079082	0.038041
4	2.5	0.016444	0.003721
5	1.5	0.016944	0.003812
5	2	0.017512	0.003234
5	2.5	0.014468	0.000744
6	1	0.017776	0.003433
6	1.5	0.013838	0.002403
6	2	0.016006	0.002989
6	2.5	0.007702	0.001197

Table S3. Comparison of the performance of the developed flexible pressure sensor unit with reported fabric-based flexible pressure sensors.

Sensitivity	Work life	Response time (ms)	Materials	Ref.
6.31 kPa^{-1} (0-150 kPa)	2000	300/260	Mxene/nonwoven fabric	[25]
5.3 kPa^{-1} (0-1.3 kPa), 2.27 kPa^{-1} (1.3-10.25 kPa), 0.57 kPa^{-1} (10.25-40.73 kPa), 0.08 kPa^{-1} (40.73-160 kPa)	1000	50/20	Mxene/cotton fabric	[26]
2.77 kPa^{-1} (0-0.2 kPa), 0.212 kPa^{-1} (2-20 kPa), 0.0042 kPa^{-1} (250-500 kPa)	-	40/80	RGO/carbonized cellulose fabric	[27]
0.016 kPa^{-1} (0-5 kPa), 0.1891 kPa^{-1} (5-30 kPa)	5000	50/90	Mxene/cotton/spandex fabric	[28]
0.168 kPa^{-1} (0-95 kPa), 0.023 kPa^{-1} (95-190 kPa)	2000	30/41	AgNWs/Mxene/fabric	[29]
2.32 kPa^{-1} (0-31 kPa), 1.26 kPa^{-1} (31-73 kPa), 0.57 kPa^{-1} (73-120 kPa)	2000	300/160	PDMS/AgNWs/MXene/fabric	[30]

0.4062 kPa^{-1} (40-240 kPa), 0.1893 kPa^{-1} (240-300 kPa)	5000	104/162	Bacterial cellulose/polypyrrole/spacer fabric	[31]
3.67 kPa^{-1} (0-20 kPa), 10.03 kPa^{-1} (20-118 kPa)	2000	135/100	MWCNT/perfluorodecyltrichlorosilane	[32]
80.79kPa^{-1} (2.1-8.2 kPa), 11.03 kPa^{-1} (8.2-13 kPa)	12500	40/85	wool felt/Mxene/fabric	[33]
80.8kPa^{-1} (0-5 kPa), 31.2 kPa^{-1} (5-12 kPa)	4000	150	SWCNTs/PVP/rGO	[34]
24.4kPa^{-1} (0.94-9.4 kPa), 6.95 kPa^{-1} (18.8-141 kPa)	5000	-	Mxene/cellulosic nonwoven textiles	[35]
0.07098kPa^{-1} (0-6 kPa), 0.00547 kPa^{-1} (6-3380 kPa)	4000	100/80	PI fiber/FCNT	[36]
**0.28kPa^{-1} (29.765-1028.717 kPa), 1 kPa^{-1} (1028.717-2288.53 kPa), 2.68 kPa^{-1} (2288.53-3770.902 kPa)**	**30000**	**10.3/3.4**	**graphene/polyester-cotton**	**This work**

Table S4. Detailed architecture of the developed pose classification model.

Layer	Output shape	Parameters
Conv1d	[n,256,18]	24832
Conv1d	[n,512,18]	393728
Conv1d	[n,1024]	1573888
Adaptive average pooling	[n,1024,1]	0
Flatten	[n,1024]	0
Linear	[n,2048]	2099200
Linear	[n,1024]	2098176
Linear	[n,10]	10250
Total		6200074

Table S5. Detailed architecture of the developed pose estimation model.

Layer	Output shape	Parameters
Linear	[n,120,128]	4224
Multi-head attention	[[n,2,128], [n,120,120]]	0
Layer normalization	[n,2,128]	256
Linear	[n,2,512]	66048
Linear	[n,2,128]	65664
Layer normalization	[n,2,128]	256
Transformer encoder layer	[n,2,128]	0
Multi-head attention	[[n,2,128], [n,120,120]]	0
Layer normalization	[n,2,128]	256
Linear	[n,2,512]	66048
Linear	[n,2,128]	65664
Layer normalization	[n,2,128]	256
Transformer encoder layer	[n,2,128]	0
Multi-head attention	[[n,2,128], [n,120,120]]	0
Layer normalization	[n,2,128]	256
Linear	[n,2,512]	66048
Linear	[n,2,128]	65664
Layer normalization	[n,2,128]	256
Transformer encoder layer	[n,2,128]	0
Multi-head attention	[[n,2,128], [n,120,120]]	0
Layer normalization	[n,2,128]	256
Linear	[n,2,512]	66048
Linear	[n,2,128]	65664

Layer normalization	[n,2,128]	256
Transformer encoder layer	[n,2,128]	0
Transformer encoder	[n,2,128]	0
Linear	[n,256]	33024
Linear	[n,512]	131584
Linear	[n,256]	131328
Linear	[n,18]	4626
Total		833682

Movie S1. Comparative test results showcasing the model-predicted joint positions with joint positions extracted from recorded video.
